# Type of childhood maltreatment and the risk of criminal recidivism in adult probationers: a cross-sectional study

**DOI:** 10.1186/s12888-016-1001-8

**Published:** 2016-08-19

**Authors:** Eun Young Kim, Jiung Park, Bongseog Kim

**Affiliations:** Department of Psychiatry, Inje University Sanggye Paik Hospital, Dongil-ro 1342, Nowon-gu Seoul, 139-707 Republic of Korea

**Keywords:** Types of childhood maltreatment, Criminal recidivism, Adult probationers, Mental-health problem

## Abstract

**Background:**

Childhood maltreatment is strongly associated with delinquency and the repeated crime. Specific types of childhood maltreatment have been found to have differential effects on recidivism in juvenile offenders, but studies of adult probationers have not been performed. This study investigated the relationship between having a history of childhood maltreatment and mental-health problems and the independent contribution of specific types of maltreatment and mental-health problems to the criminal recidivism of adult probationers.

**Method:**

This study included 183 adult probationers (107 males and 76 females) with a mean age of 40.1 (SD = 11.8) years. Type of childhood maltreatment was assessed using the Childhood Trauma Questionnaire, which consists of five subscales (emotional neglect and abuse, physical neglect and abuse, and sexual abuse). Additionally, we used the Mini International Neuropsychiatric Interview to assess participants for the presence of psychiatric disorders and assessed levels of emotional dysregulation and resilience. Hierarchical logistic regression analysis was performed to determine whether the types of childhood maltreatment were independently associated with repeated crime, after adjusting for demographic factors and mental-health problems.

**Results:**

The overall prevalence of mental illness in the childhood maltreatment group was significantly higher than in the no childhood maltreatment group (56.1 % vs. 38.2 %, *p* = 0.017). The maltreated group had a higher rate of major depressive disorder, a higher level of emotional dysregulation, and a lower level of resilience than the group that was not maltreated. Recidivism was uniquely associated with physical neglect (Adjusted Odds Ratio [AOR], 2.862; 95 % Confidence Interval [95 % CI], 1.213–6.752) and the presence of at least one psychiatric disorder (AOR, 3.791; 95 % CI, 1.703–8.443).

**Conclusions:**

Childhood maltreatment deserves further attention in adult probationers because it is potentially associated with higher rates of psychiatric morbidity and recidivism. In particular, physical neglect during childhood plays a critical role in repeated crime, independent of mental-health problems for high-risk adults involved with the criminal justice system. Rigorous evaluations of the relevance of childhood maltreatment in the assessment and treatment of criminal offenders are needed.

## Background

Childhood maltreatment has pronounced negative effects on mental health. It leads to disruptions in interpersonal relationships and psychological problems, including low self-efficacy, lack of positive task orientation, and social information processing deficits [1, 2], and it interferes with the development of emotional self-regulation [3]. It is not surprising that child maltreatment has also been linked to a variety of psychiatric disorders and mental-health problems in adulthood, such as suicidality, depression, substance abuse, psychotic disorders, and the perpetration of violence. Although the mechanism linking childhood maltreatment and adverse mental-health outcomes has remained unclear, it is known that childhood maltreatment influences brain maturation and serotonergic functioning [4–7]. Serotonergic function is crucial for the regulation of emotional responses, impulsivity, and aggression [8, 9]. Possibly, this epigenetic change leads to the perpetuation of poor mental-health outcomes and social adjustment in adults, as has been emphasized in studies of psychiatric patients as well as the general population [10–14].

One of the most detrimental consequences of childhood maltreatment is that victims are likely to exhibit several socio-behavioral difficulties, and subsequently become perpetrators of crime. A substantial number of studies have suggested that childhood maltreatment significantly increases the risk of criminality in adolescence and adulthood. Prior prospective studies have reported that maltreated children are 2 – 6 times more likely to develop criminal behavior in young adulthood, compared to non-maltreated controls [15–18]. For example, a study exploring the relationship between child maltreatment and criminal behavior over three developmental periods (i.e., adolescence, early adulthood, adulthood) in the National Youth Survey, found that childhood maltreatment was significantly associated with involvement in criminal behavior that continued into adulthood across crime types, including index offenses and intimate partner violence [19]. Given that childhood maltreatment is strongly associated with delinquent behavior, there is sufficient evidence to expect childhood maltreatment to be associated with recidivism [10, 20, 21]. Moreover, it has been suggested that the specific types of childhood maltreatment (i.e. abuse and neglect) might have differential effects on recidivism, and that neglect might be a stronger predictor of recidivism than the other different types of abuse in adolescence, after controlling for a wide range of family, peer, academic, mental health, and substance-abuse covariates [22–24]. These studies have been facilitated in recent years by the introduction of measures of different types of trauma, such as the Childhood Trauma Questionnaire (CTQ), which provides a more sensitive measure of abuse and neglect than official records [25].

Although many investigations have focused on the association between childhood maltreatment and recidivism, most of the findings have been reported in studies of juvenile offenders or specific clinical populations, such as Attention-Deficit/Hyperactivity Disorder (ADHD) [10, 22–24, 26]. Furthermore, few studies have investigated the relationship between child maltreatment and criminal recidivism in high-risk populations, such as probationers. Probationers are responsible for less severe and destructive crimes and lead their lives in the community under the supervision of the justice system. As such, they might be the most relevant target population for interventions to prevent recidivism, promote social adjustment, manage underlying psychosocial problems, and ultimately minimize the impact of crimes on individuals, families, and society. The identification of probationers’ risk factors for recidivism has clinical and policy implications for the development of risk-management interventions for repeat offenders.

The aim of this study was to investigate: (1) the relationships between a history of childhood maltreatment and mental-health problems, and (2) the independent contribution of specific types of maltreatment and mental-health problems to recidivism, based on epidemiological data of adult probationers in South Korea. Given the findings discussed previously, we hypothesized that specific types of childhood maltreatment would be associated with an increased risk of recidivism and that maltreatment would independently contribute to the risk, over other mental-health problems.

## Methods

### Participants

This study was conducted with a sample of adult probationers supervised by one probation office in Seoul between August and November in 2014. A total of 206 adult probationers agreed to participate in face-to-face interviews and complete a self-report questionnaire. Information regarding demographic characteristics (gender, age), living situation (living alone, living together), educational level, and crime-related data (including type of criminal offense and recidivism), was obtained using self-administered questionnaires.

### Assessment of childhood maltreatment

For the purposes of this study, we divided the sample into two groups: adult probationers with and without a history of childhood maltreatment. Childhood maltreatment was assessed using the Korean version of the CTQ, which demonstrated good psychometric properties in a study with a Korean sample (Cronbach’s alpha = 0.79) [27]. The CTQ consists of 28 items: five subscales consisting of 5 items each, measure emotional neglect, emotional abuse, physical neglect, physical abuse, and sexual abuse; and one 3-item subscale measures minimization/denial [28]. Emotional abuse refers to verbal assaults on a child’s sense of worth or well being, or any humiliating, demeaning, or threatening behavior directed toward a child by an older person. Physical abuse refers to bodily assaults on a child by an older person that pose a risk of, or result in, injury. Sexual abuse refers to sexual contact or conduct between a child and an older person, including explicit coercion. Emotional neglect refers to the failure of caretakers to provide basic psychological and emotional needs, such as love, encouragement, belonging and support. Physical neglect refers to failure to provide basic physical needs including food, shelter, and safety. The Minimization/Denial scale is used to detect underreporting of traumatic events. Each item is scored on a 5-point Likert scale with higher scores indicating a more severe degree of childhood maltreatment. In the current study, the cutoff scores for moderate to severe maltreatment for each subscale were the same as those used in previous studies (>14 for emotional neglect, > 12 for emotional abuse, > 9 for physical neglect and abuse, and > 7 for sexual abuse) [10, 29–31]. The individuals who met the criteria for moderate to severe levels on one or more of the subscales were categorized as probationers with a history of childhood maltreatment.

### Diagnosis of psychiatric disorders

In the present study, a clinical psychologist used the Mini International Neuropsychiatric Interview (MINI), a structured interview designed to detect a wide range of DSM-IV and ICD-10 mental disorders. The MINI has been used to conduct a comprehensive assessment of mental illness and suicidality in several studies in criminal justice settings [32, 33]. The MINI has been used in more than 45 nations worldwide, and was translated into Korean and validated in 2006 [34]. The overall agreement between the Korean version of the MINI and diagnoses by experts was high, and the range of the Kappa values was 0.22 (somatoform disorder) to 0.93 (bipolar disorder-past). The Kappa values were high for major depressive disorder (0.71), bipolar disorder [0.74 (current) to 0.93 (past)], anxiety disorders (0.62–0.81), alcohol dependence (0.77), and schizophrenia [0.41 (past) to 0.69 (current)]. The Kappa values were relatively low for somatoform disorder (0.22), delusional disorder (0.33), and dysthymia (0.34) [34]. For the disorders not assessed by the MINI, such as gambling disorder and internet game disorder, we conducted the interviews using DSM-5 criteria. We also administered the Adult ADHD Self-Report Scale for ADHD, which was developed based on the DSM-IV diagnostic criteria for ADHD by the World Health Organization [35]. In this study, six of the scale’s questions that are known to be the best predictors of ADHD symptoms were used. The frequency of experiencing symptoms for the last six months was measured for each question, and the wording and phrasing of the questions were tailored for use with adults.

### Assessment of resilience and emotional dysregulation

We used the Korean Version of the Difficulties in Emotion Regulation Scale (K-DERS) and the Korean Version of the Connor-Davidson Resilience Scale (CD-RISC) to assess participants’ mental status [36, 37]. The CD-RISC is a 25-item measure used to assess resilience. Resilience may be viewed as a measure of the ability to cope with stress [38]; it may be further defined as a method for overcoming negative circumstances or adapting effectively to traumatic encounters, and evading the negative consequences associated with risk [39]. CD-RISC items are rated on a 5-point scale, ranging from 0 to 4. A higher total score indicates higher resilience. The CD-RISC has been reported to have the best psychometric properties among the measures of resilience [40]. The K-DERS is a 36-item, multidimensional self-report instrument that measures emotional dysregulation, which refers to difficulties within the following dimensions of emotion regulation: (a) an awareness and understanding of emotions; (b) the acceptance of emotions; (c) the ability to engage in goal-directed behavior and refrain from impulsive behavior when experiencing negative emotions; and (d) access to emotion regulation strategies perceived as effective [41]. Items are rated on a 5-point scale ranging from 1 (almost never) to 5 (almost always). A higher total score indicates a higher level of emotional dysregulation. Research has confirmed the reliability and validity of the K-DERS and CD-RISC [36, 37].

### Statistical analyses

The socio-demographic and clinical variables of the two groups (i.e., the adult probationers with and without a history of childhood maltreatment) were compared using Student’s *t*-test for continuous measures and the chi-square test for categorical measures. In addition, we assessed whether the different types of childhood maltreatment were independently associated with repeated crime, using hierarchical multivariate logistic regression analysis. Demographic factors (age, gender, educational level, and living situation) were entered at Step 1 and mental-health factors (diagnosis of at least 1 psychiatric disorder, emotional dysreguation, and level of resilience) were entered at Step 2. Finally, the specific types of childhood maltreatment were entered at Step 3. All analyses were performed using SAS Enterprise 4.2 (SAS Institute Inc., Cary, North Carolina, USA), and *p* < .05 was considered statistically significant.

## Results

### Socio-demographic and clinical characteristics

Among the 206 participants, 7 did not complete the CTQ, and 16 did not complete the Minimization/Denial scale of the CTQ; thus, they were excluded from the analysis. Of the final 183 participants aged 19 – 74, the average age was 40.1 (SD = 11.8) years and the majority of them were male (83.6 %). We found that 107 (58.5 %) participants reported more than one type of childhood maltreatment according to their responses to the CTQ. The frequencies of emotional neglect, emotional abuse, physical neglect, physical abuse, and sexual abuse were 24 %, 7.1 %, 39.9 %, 24 %, and 12 %, respectively.

Table 1 presents the socio**-**demographic characteristics of the population according to the presence of history of childhood maltreatment. There was no significant difference between the childhood maltreatment group and no childhood maltreatment group with regard to gender, age, or living situation. However, there were significantly greater numbers of probationers in the childhood maltreatment group who had lower levels of education. In the childhood maltreatment group, the rates of the different types of criminal offenses were as follows: 23.1 % for property crimes, 21.2 % for domestic violence, 16.3 % for violent crimes, 14.4 % for sexual crimes, and 12.5 % for drug crimes. For the no childhood maltreatment group, the rates were as follows: 22.7 % for property crimes, 12 % for domestic violence, 13.3 % for violent crimes, 14.7 % for sexual crimes, and 14.7 % for drug crimes. In this group, the rate of domestic violence was ranked fifth, whereas it was ranked second in the childhood maltreatment group. However, there was no statistically significant difference with regard to type of crime between the two groups.

**Table 1:** Socio-demographic characteristics of participants with and without childhood maltreatment

Variables	Childhood maltreatment group (*n* = 107)	No childhood maltreatment group (*n* = 76)	*P*-value
Childhood Trauma Questionnaire, mean ± SD	47.4 ± 8.4	31.0 ± 4.6	<0.001
Number of types of maltreatment, N (%)^a^			
1	53 (29.0)		
2	32 (17.5)		
3	12 (6.6)		
4	7 (3.8)		
5	3 (1.6)		
Age (years), mean ± SD	40.1 ± 12.7	40.1 ± 10.5	0.978
Male, N (%)	92 (86.0)	61 (80.3)	0.303
Living situation, N (%)			
Living alone	27 (26.7)	14 (19.2)	0.247
Living together	74 (73.3)	59 (80.8)	
Education, N (%)			
< high school graduation, N (%)	23 (22.1)	11 (14.7)	0.010
college graduation	54 (51.9)	28 (37.3)	
> college graduation	27 (26.0)	36 (48.0)	
Crime type, N (%)			
Violent crime	17 (16.3)	10 (13.3)	0.578
Property crime	23 (22.1)	17 (22.7)	0.930
Sexual crime	15 (14.4)	11 (14.7)	0.964
Drug crime	13 (12.5)	11 (14.7)	0.675
Domestic violence	22 (21.2)	9 (12.0)	0.110
Traffic offences	1 (1.0)	3 (4.0)	0.175
Obstruction of justice	2 (1.9)	3 (4.0)	0.405
Drunk driving	8 (7.7)	5 (6.7)	0.794
Others	5 (4.8)	6 (8.0)	0.380

^a^based on cutoff score for moderate to severe trauma in each type in Korean Childhood Trauma Questionnaire

*Note: SD* Standard deviation

### Childhood maltreatment and adult mental-health variables

Table 2 presents the prevalence of psychiatric disorders and other psychological factors such as emotional dysregulation and level of resilience by childhood maltreatment. The overall prevalence of mental illness in the childhood maltreatment group was 56.1 %, and it was significantly higher than in the no childhood maltreatment group (38.2 %) (*p* = 0.017). Major depressive disorder was significantly more prevalent in the childhood maltreatment group (13.1 % vs. 1.3 %, *p* = 0.004). There were no significant differences with respect to the other mental disorders, including alcohol use disorder, which was the most prevalent disorder in both groups (31.8 % and 28.9 % respectively). Compared to the no childhood maltreatment group, the childhood maltreatment group had a significantly higher K-DERS score and lower CD-RISC score (*p* < 0.001 on both measures). Compared to the no maltreatment group, the childhood maltreatment group’s MINI and suicidality scores were significantly higher (*p* = < 0.001 on both measures).

**Table 2:** Mental health problems in participants with and without childhood trauma

	Childhood maltreatment group (*n* = 107)	No childhood maltreatment group (*n* = 76)	*P*-value
Psychiatric diagnoses, N (%)			
*At least one diagnosis*	60 (56.1)	29 (38.2)	0.017
Major depressive episode	14 (13.1)	1 (1.3)	0.004
Dysthymic disorder	3 (2.8)	1 (1.3)	0.498
Manic/Hypomanic episode	7 (6.5)	5 (6.6)	0.992
Panic disorder	1 (0.9)	0 (0.0)	0.398
Agoraphobia	1 (0.9)	0 (0.0)	0.398
Social phobia	1 (0.9)	1 (1.3)	0.807
Obsessive compulsive disorder	3 (2.8)	2 (2.6)	0.944
Posttraumatic stress disorder	2 (1.9)	1 (1.3)	0.771
Alcohol use disorder	34 (31.8)	22 (28.9)	0.782
Substance use disorder	3 (2.8)	2 (2.6)	0.944
Psychotic disorder	4 (3.7)	1 (1.3)	0.322
Anorexia nervosa	0 (0.0)	0 (0.0)	
Bulimia nervosa	5 (4.7)	2 (2.6)	0.478
Generalized anxiety disorder	3 (2.8)	1 (1.3)	0.498
Antisocial personality disorder	7 (6.5)	1 (1.3)	0.088
Pathologic gambling	6 (5.6)	1 (1.3)	0.136
Internet gaming disorder	4 (3.7)	0 (0.0)	0.088
ADHD^a^	5 (4.7)	1 (1.3)	0.209
Suicidality score, Mean ± SD^b^	4.36 ± 8.36	0.66 ± 2.27	<0.001
K-DERS, Mean ± SD	73.80 ± 20.48	62.01 ± 19.62	<0.001
CD-RISC, total, Mean ± SD	62.88 ± 19.58	74.82 ± 15.98	<0.001

^a^ based on Adult ADHD Self-Report Scale

^b^ Suicidality score based on Mini International Neuropsychiatric Interview

*Note: ADHD* Attention-Deficit/Hyperactivity Disorder, *CD-RISC* Connor-Davidson Resilience Scale, *K-DERS* Korean version of Difficulties in Emotion Regulation Scale, *SD* Standard deviation

### Association between specific types of childhood maltreatment and recidivism

The contribution of specific types of childhood maltreatment to recidivism was assessed using hierarchical logistic regression analysis (Table 3). The mental-health factors resulted in a significant improvement in the prediction of recidivism (*χ*^*2*^(3) = 13.66, *p* = 0.007). Among the mental-health factors, only the presence of psychiatric disorders was significantly related to recidivism (Adjusted Odds Ratio [AOR], 3.791; 95 % Confidence Interval [95 % CI], 1.703–8.443). Finally, the types of childhood maltreatment also significantly improved the predictive power for recidivism (*χ*^*2*^(5) = 11.37, *p* = 0.001). Recidivism was uniquely associated with physical neglect (AOR, 2.862; 95 % Confidence Interval [95 % CI], 1.213–6.752) and the presence of at least one psychiatric disorder (AOR, 3.791; 95 % CI, 1.703–8.443). We performed additional logistic regression analyses, which included the specific psychiatric disorders listed in Table 2, instead of presence of at least 1 psychiatric disorder. In these models, we found that major depressive disorder, bipolar disorder, and alcohol use disorder were significantly related to recidivism as well as physical neglect. (all *p*s < 0.05).

**Table 3:** Hierachical logistic regression analysis predicting recidivism from different types of childhood maltreatment and mental health problems

Variables	Step 1	Step 2	Step 3
Demographic variables			
Age	1.009 (0.980-1.040)	1.027 (0.993-1.062)	1.027 (0.991-1.064)
Gender	0.599 (0.218-1.644)	0.581 (0.200-1.690)	0.575 (0.186-1.776)
Living alone	0.559 (0.259-1.206)	0.456 (0.199-1.044)	0.462 (0.187-1.146)
Education level	0.746 (0.454-1.225)	0.759 (0.443-1.302)	0.909 (0.508-1.627)
Mental Health problem			
At least one psychiatric disorder		3.791 (1.703-8.443)**	4.257 (1.795-10.079)**
K-DERS		0.980 (0.958-1.002)	0.976 (0.952-1.000)
CD-RISC		0.983 (0.961-1.007)	0.991 (0.967-1.016)
Types of childhood maltreatment			
Emotional neglect			1.845 (0.745-4.571)
Emotional abuse			0.792 (0.163-3.853)
Physical neglect			2.862 (1.213-6.752)**
Physical abuse			0.753 (0.281-2.021)
Sexual abuse			1.611 (0.541-4.795)
-2 log likelihood ratio	195.994	182.336	170.962
Δχ^2^(df)		13.659 (3)**	11.373 (5)**
ΔR^2^		0.111	0.186

*Note:* *p < .05, **p < .01, ***p < .001, *CD-RISC* Connor-Davidson Resilience Scale, *K-DERS* Korean version of Difficulties in Emotion Regulation Scale

## Discussion

The main objectives of this study were to examine the relationship between a history of childhood maltreatment and the development of later mental-health problems, and to examine the unique contribution of specific types of child maltreatment to the prediction of criminal recidivism, over mental-health problems in adult probationers. The study’s results indicated that childhood maltreatment was significantly associated with psychiatric disorders, especially a major depressive episode, and with lower resilience and higher emotional dysregulation. Among the types of childhood maltreatment, physical neglect was significantly related with the recidivism, even after controlling for the presence of psychiatric disorders. Our finding of the association between physical neglect and recidivism is consistent with prior studies, but is, to our knowledge, the first demonstration of this relationship among adult probationers. This finding may clarify the risk profile to guide interventions targeting desistance from crime in high-risk populations (i.e. probationers).

The rate of childhood maltreatment in this study’s adult probationers was 58.5 %, which is higher than the rate in the general population; the rates of childhood maltreatment were reported to be 24–32 % globally [42, 43] and 40.8 % in a South Korean study using the same CTQ cut-off score [30]. Among the types of childhood maltreatment, emotional neglect and physical neglect were 24 % and 39.9 % respectively, which is far higher than what has been found in healthy adults (14.6 % and 13.6 %, respectively). The other types of childhood maltreatment rates in our sample of probationers are comparable to the rates reported in a previous study [30]. Therefore, an overall higher rate of childhood maltreatment in adult probationers may result from a higher rate of neglect victimization, compared to the general population.

In this study, childhood maltreatment was associated with psychiatric morbidity, especially major depressive disorder, as well as low resilience and a high degree of emotional dysregulation. Overall, these results are consistent with other studies showing that childhood maltreatment is associated with poor mental health and social adjustment [12–14, 44]. The higher prevalence of major depressive disorder and suicidality in adult probationers with a history of childhood maltreatment was in agreement with previous studies [14, 45–48]. However, we could not find any significant differences in the prevalence of each mental disorder between groups, including alcohol use disorder, which was possibly due to the small sample size.

Our results identified physical neglect as an independent predictor of criminal recidivism, after controlling for mental-health problems, which is generally in agreement with the results of previous studies. Childhood neglect has long been considered to be a significant predictor of criminal behavior [13, 16, 49, 50]. However, many prior studies included only youths and they have categorized “neglect” as one type of maltreatment, rather than dividing it into “physical neglect” and “emotional neglect” with different measures to assess childhood trauma, instead of the CTQ [22, 24, 26]. Dembo et al. found that neglect was a stronger predictor of recidivism than both physical and sexual abuse in juvenile offenders [22]. Recently, a study found that neglect victimization was uniquely related to general recidivism, whereas physical abuse victimization was uniquely related to violent recidivism among male juvenile offenders, over and above the dynamic risk factors for recidivism [26]. However, among female juvenile offenders, none of the types of maltreatment were uniquely related to general or violent felony recidivism [26]. Although most of the participants included in our study consisted of males, our results were similar to that study. The results of our study contribute to the literature of adult probationers by showing the independent relationship between victimization by physical neglect and recidivism after controlling for several mental-health factors.

Interestingly, our results show the unique association between recidivism and physical neglect, but not with emotional neglect. To date, few studies have investigated the association between specific types of childhood neglect defined by the CTQ and criminal recidivism. In contrast to our results, Kingree at al. revealed that relatively high levels of emotional neglect, but not physical neglect, were associated with recidivism after controlling for single and multiple episodes of recidivism and the simultaneous influence of different socio-demographic, behavioral, and maltreatment variables among adolescent detainees [23]. They stated that a conclusion should not be drawn from their data as to why emotional neglect and physical neglect were associated with recidivism in opposite ways. However, the discrepancy in the results might be partly due to cultural factors and differences in the study population. The CTQ defines physical neglect based on whether respondents had enough to eat, if their parents’ drinking interfered with their care, if they ever wore dirty clothes, and if there was someone to take them to a doctor. Physical neglect can be considered a failure to provide basic physical safety and security, including food, shelter, and clothing, which comprise the traditional list of immediate basic needs [51]. On the other hand, emotional neglect can be defined based on whether the family made the respondent feel special and loved and if the family was a source of strength, support, and protection [25]. Compared to physical neglect, the construct of emotional neglect can be operationalized differently across cultures because it is associated with personal perceptions of caregiver-child interactions. Additionally, child-rearing practices, such as parenting attitudes and parent-child relationships are very different across cultures. East Asian countries emphasize the establishment of both physical and emotional closeness so that a lifelong bond is assured and the maintenance of parental authority and children’s obedience through harsh discipline [52]. In this context, the people of East Asia might have higher expectations of parent-child emotional interactions through their development, compared with those of Western cultures. They might also have a different view of an ideal caregiver-child interaction. All these factors may influence the evaluation of emotional neglect using the CTQ, and subsequently, its effect on recidivism. The differential effects of physical and emotional neglect on repeated crime and their possible moderating factors should be explored in future studies from a cross-cultural perspective. On the other hand, these differences might be related to the study population’s age. Whereas most of the previous studies have included adolescents, our study consisted of adults. The developmental consequences of adversity are neither restricted to the particular developmental period in which they occur, nor do they reflect a linear relationship between adversity and outcomes in adulthood. The processes underlying the consequences of childhood adversity are best viewed as developmental cascades, or the (nonlinear) cumulative consequences of interactions across multiple levels (i.e., brain to behavior) and contexts (i.e., internal and external environments) over one’s entire life [53].

The present study has several limitations. First, the CTQ is a self-report questionnaire, which may raise a recall bias issue because responses might be incorrect due to forgetting, and/or the influence of mood at the time of its completion. For example, those who are mentally distressed may be more likely to recall their childhood as adverse. Second, crime data were obtained from a self-report questionnaire, which might be different from respondents’ actual criminality, making the reports less reliable than official data on the person’s criminal history. Third, this study uses a cross-sectional design, so a causal relationship between childhood maltreatment and recidivism cannot be inferred. A prospective longitudinal analysis with a larger sample will be necessary, controlling for socioeconomic status, psychiatric morbidity, and dynamic, modifiable risk factors.

## Conclusions

The data presented in our study suggest childhood maltreatment of adult probationers should receive more attention because it is potentially associated with higher rates of psychiatric morbidity, suicidality, and recidivism. In particular, physical neglect during childhood plays a critical role in repeated crime, independent of mental-health problems, for high risk adults involved with the criminal justice system. Our results provide useful information for clinical practice and policy development to promote social adjustment in adult probationers and help them desist from the crime. Rigorous evaluations of the relevance of childhood maltreatment victimization in the assessment and treatment of criminal offenders are needed.

## Abbreviations

ADHD: attention-deficit/hyperactivity disorder
AOR: adjusted odds ratio
CD-RISC: connor-davidson resilience scale
CTQ: childhood trauma questionnaire
K-DERS: Korean version of the difficulties in emotion regulation scale
MINI: mini international neuropsychiatric Interview
